# Capturing an Early Gene Induction Event during Wood Decay by the Brown Rot Fungus *Rhodonia placenta*

**DOI:** 10.1128/aem.00188-22

**Published:** 2022-03-29

**Authors:** Claire E. Anderson, Jiwei Zhang, Lye Meng Markillie, Hugh D. Mitchell, William B. Chrisler, Matthew J. Gaffrey, Galya Orr, Jonathan S. Schilling

**Affiliations:** a Bioproducts and Biosystems Engineering, University of Minnesota, St. Paul, Minnesota, USA; b Environmental Molecular Science Laboratory, Pacific Northwest National Laboratory, Richland, Washington, USA; c Biological Sciences Division, Pacific Northwest National Laboratory, Richland, Washington, USA; d Plant and Microbial Biology, University of Minnesota, St. Paul, Minnesota, USA; Nanjing Agricultural University

**Keywords:** decomposition, lignocellulose, biodegradation, RNA, transcriptomics

## Abstract

Brown rot fungi dominate wood decomposition in coniferous forests, and their carbohydrate-selective mechanisms are of commercial interest. Brown rot was recently described as a two-step, sequential mechanism orchestrated by fungi using differentially expressed genes (DEGs) and consisting of oxidation via reactive oxygen species (ROS) followed by enzymatic saccharification. There have been indications, however, that the initial oxidation step itself might require induction. To capture this early gene regulation event, here, we integrated fine-scale cryosectioning with whole-transcriptome sequencing to dissect gene expression at the single-hyphal-cell scale (tens of micrometers). This improved the spatial resolution 50-fold, relative to previous work, and we were able to capture the activity of the first 100 μm of hyphal front growth by Rhodonia placenta in aspen wood. This early decay period was dominated by delayed gene expression patterns as the fungus ramped up its mechanism. These delayed DEGs included many genes implicated in ROS pathways (lignocellulose oxidation [LOX]) that were previously and incorrectly assumed to be constitutively expressed. These delayed DEGs, which include those with and without predicted functions, also create a focused subset of target genes for functional genomics. However, this delayed pattern was not universal, with a few genes being upregulated immediately at the hyphal front. Most notably, this included a gene commonly implicated in hydroquinone and iron redox cycling: benzoquinone reductase.

**IMPORTANCE** Earth’s aboveground terrestrial biomass is primarily wood, and fungi dominate wood decomposition. Here, we studied these fungal pathways in a common “brown rot”-type fungus, *Rhodonia placenta*, that selectively extracts sugars from carbohydrates embedded within wood lignin. Using a space-for-time design to map fungal gene expression at the extreme hyphal front in wood, we made two discoveries. First, we found that many genes long assumed to be “on” (constitutively expressed) from the very beginning of decay were instead “off” before being upregulated, when mapped (via transcriptome sequencing [RNA-seq]) at a high resolution. Second, we found that the gene encoding benzoquinone reductase was “on” in incipient decay and quickly downregulated, implying a key role in “kick-starting” brown rot.

## INTRODUCTION

The carbohydrate-selective, wood decay mechanisms of brown rot fungi have great appeal for biotechnology and have far-reaching implications for the global carbon cycle. Brown rot was long thought to be controlled by extracellular pH gradients (1) but was recently shown to be a two-step, sequential mechanism orchestrated by fungi using differentially expressed genes (DEGs) (2–4). Understanding how a fungus regulates this sequence may enable us to control this efficient deconstruction pathway and will help us more accurately predict carbon fluxes from wood (up to 81% of the total terrestrial biomass) (5).

During step 1 of brown rot, fungi deploy reactive oxygen species (ROS) via the Fenton reaction to initiate the depolymerization of crystalline cellulose, resulting in rapid strength loss in the plant cell wall (6, 7). The Fenton reaction (Fe^2+^ + H_2_O_2_ → Fe^3+^ + HO^−^ + HO^·^) is likely enabled by a fungus-mediated hydroquinone redox cycle, during which a hydroquinone, generated by a quinone reductase (QRD) acting on a benzoquinone, nonenzymatically reduces Fe^3+^ to Fe^2+^ (8, 9). Brown rot fungi transition to step 2 by downregulating ROS-linked genes (i.e., lignocellulose oxidation enzymes [LOXs], as previously categorized [2]) and by upregulating a limited suite of carbohydrate-active enzymes (CAZys) to hydrolyze polysaccharides (2–4). To avoid unnecessary damage to their own enzymes and hyphae, not only do brown rot fungi need to turn off ROS production as they transition to enzymatic saccharification, as previously confirmed (3), but it is logical that they also would need to upregulate relevant genes to turn on ROS production at the onset of decay. An initial inducible event has not previously been captured, but it is supported by a history of observations that brown rot fungi often fail to efficiently metabolize pure cellulose in the absence of wood (10–12).

To capture this ephemeral induction event, our approach was to use a fine-scale spatial gradient to resolve decay timing at the earliest stages of decay. We achieved a 50-fold-finer resolution than previously observed by using cryosectioning and single-cell-level transcriptomics to look at specific 100-μm wood wafer sections within the first 5 mm behind the hyphal front (approximately the first 48 h of decay) of the brown rot fungus Rhodonia placenta (2). This revealed a major induction event that was not seen at the previous lower resolution and revealed delayed expression for decay-related DEGs, including CAZys and most LOXs, and DEGs without predicted functions. These delayed DEGs collectively create a focused subset of potentially inducible, early decay genes to target discovery and assign function (see Data Set S1 in the supplemental material). However, this delayed pattern was not universal, with a few genes being upregulated immediately at the hyphal front. Most notably, this included a gene commonly implicated in hydroquinone and iron redox cycling: benzoquinone reductase.

## RESULTS

### High spatial resolution captured a major gene induction event.

**FIG 1:**
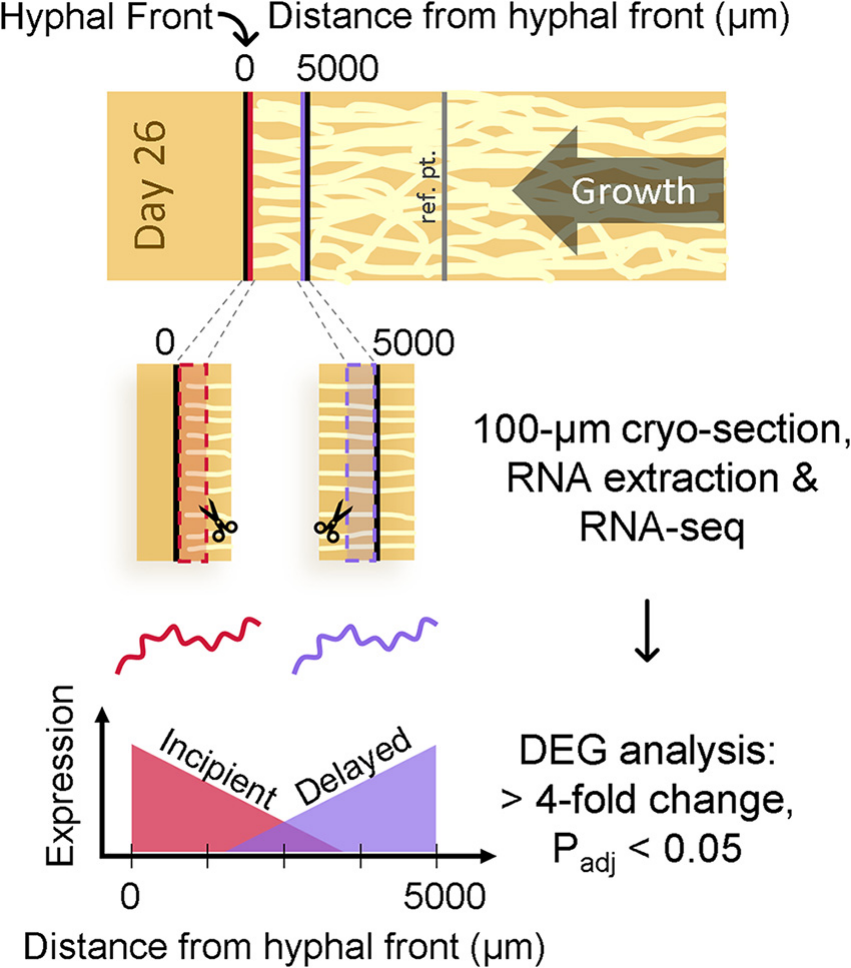
Using space to parse time at a micrometer scale. Wood wafers colonized directionally by the brown rot fungus *Rhodonia placenta* were cryosectioned to a 100-μm thickness at the hyphal front and closely behind in order to map gene expression patterns using whole transcriptomic RNA (RNA-seq). Differentially expressed genes (DEGs) with a >4-fold difference (0 to 100 μm versus 4,900 to 5,000 μm) were grouped as being downregulated after high levels near the extreme front (which we label incipient) or upregulated after a delay (delayed). An additional section was included as a reference for later decay, as shown in Fig. 4.

**FIG 2:**
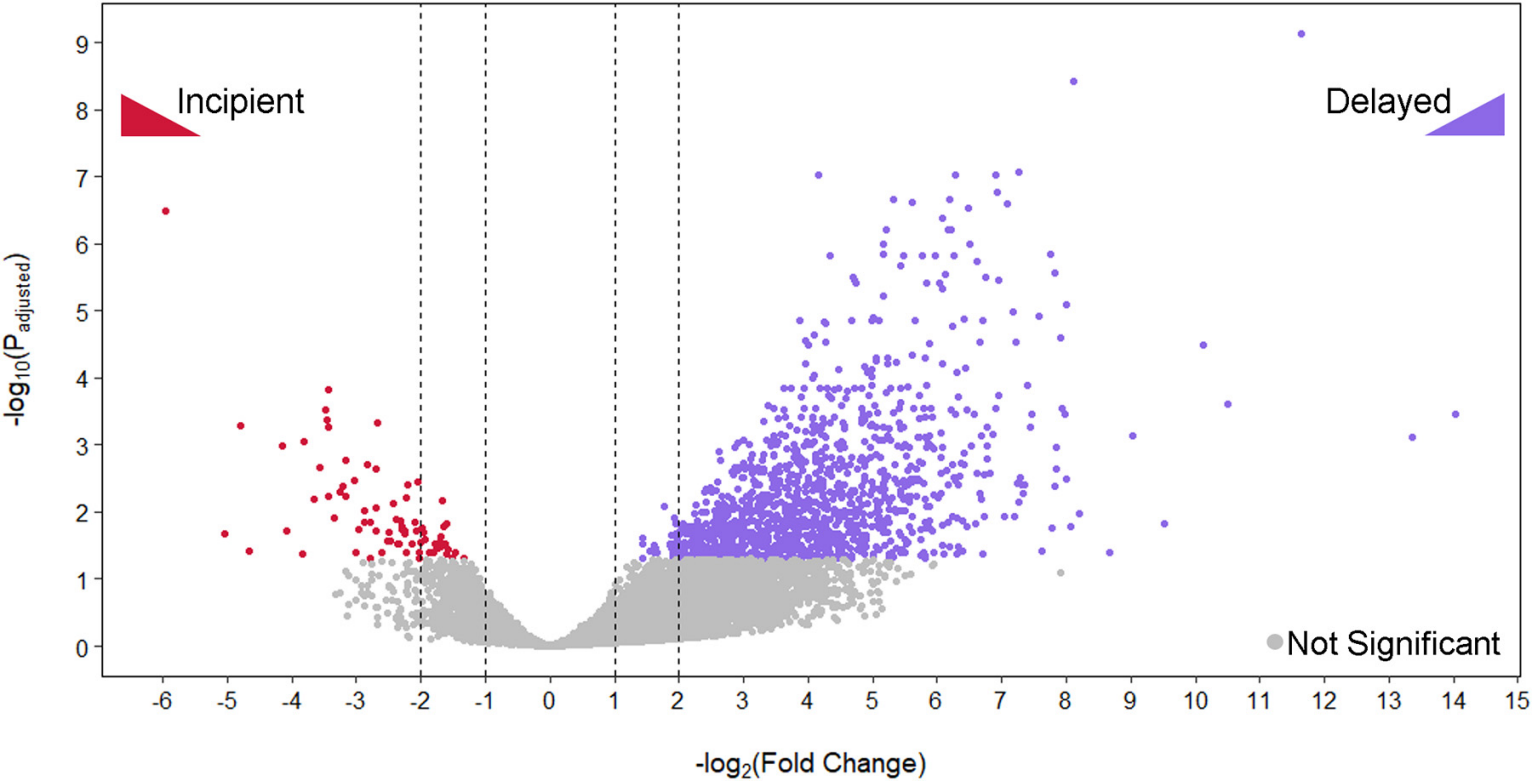
A major induction event during early wood decay by *R. placenta*. Expression patterns within the first 5 mm (∼48 h) of decay show 21-fold more delayed genes than incipient genes (*n* = 1,176 versus *n* = 56) when examining significant DEGs with a >4-fold change (i.e., dotted lines at ±2). The fold change was calculated using the ratio of 0 to 100 μm to 4,900 to 5,000 μm. Genes were considered significant if the *P*_adjusted_ value was <0.05; low-count genes (*n* = 1,668) are not displayed.

A space-for-time map of wood decay by the brown rot fungus R. placenta was enabled by physically sectioning aspen wafers colonized directionally in microcosms (2, 13) (Fig. 1). Expression patterns representing the first 48 h of decay were determined using 100-μm cryosectioning (50-fold higher than the previous 5-mm resolution [2]) and single-cell-level transcriptomics to evaluate whole-transcriptome expression at the hyphal front (0 to 100 μm; approximately hour 1) and at the 5-mm distance (4,900 to 5,000 μm; approximately hour 48). Analysis of DEGs (>4-fold change, with the fold change calculated using the ratio of 0 to 100 μm to 4,900 to 5,000 μm [adjusted *P* {*P*_adjusted_} < 0.05]) revealed a major induction event with 21-fold more DEGs being upregulated in 4,900 to 5,000 μm (which we label delayed) than in 0 to 100 μm (incipient) (Fig. 2).

### Carbohydrate-active enzyme genes followed the “rules” of delayed expression.

**FIG 3:**
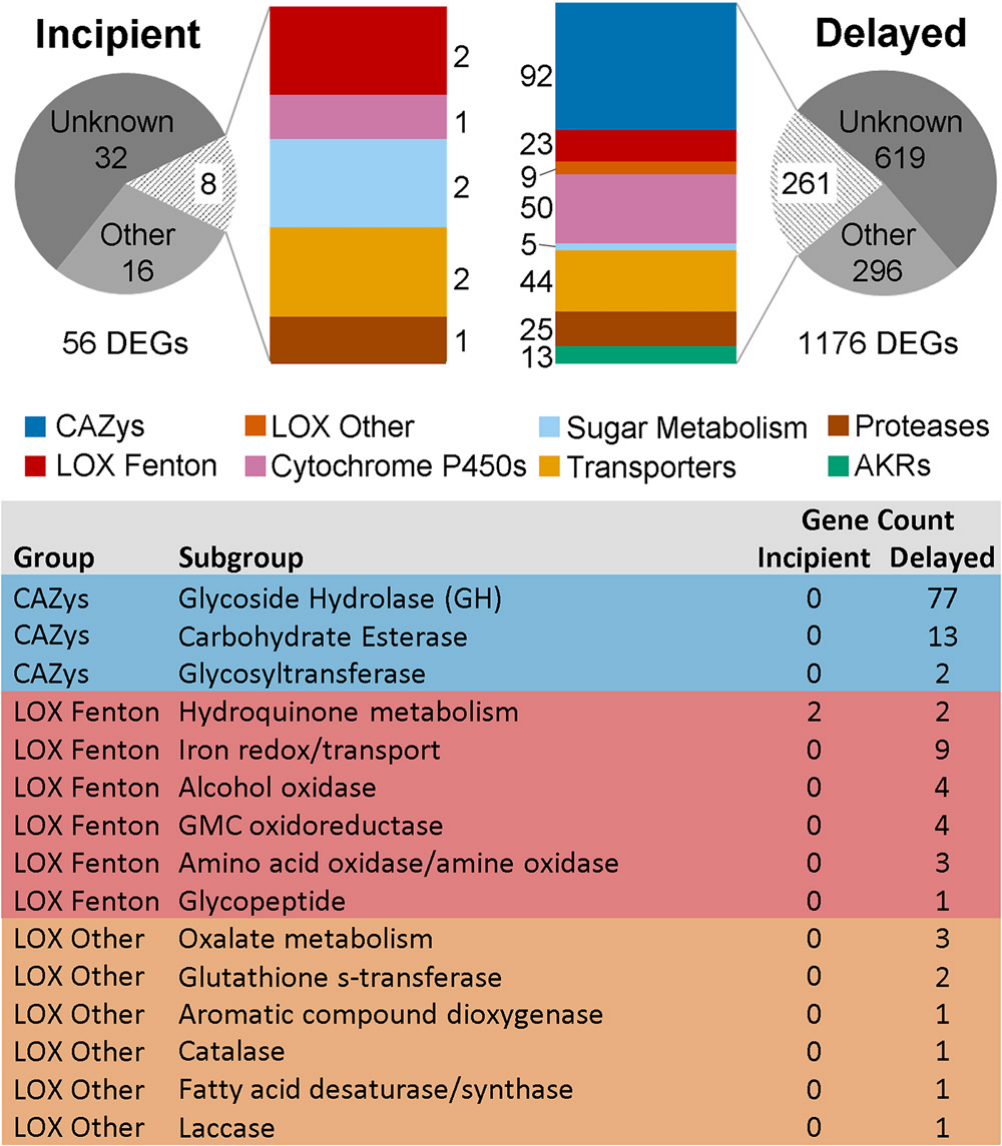
Distribution of incipient versus delayed *R. placenta* DEGs involved in deconstructing lignocellulose. Total DEGs (>4-fold [*P*_adjusted_ < 0.05]) for incipient and delayed expression are organized by categories of interest. Here, functional assignments were adopted from those described previously by Zhang et al. (2), in which carbohydrate-active enzymes (CAZys) follow definitions from the Carbohydrate Active Enzymes database (http://www.cazy.org/) (14), including glycoside hydrolases (GHs), carbohydrate esterases, and glycosyltransferases, except for enzymes with auxiliary activities (e.g., peroxidases and oxidases, etc.), which are in the lignocellulose oxidation enzyme (LOX) categories. AKR, aldo-keto reductase.

**FIG 4:**
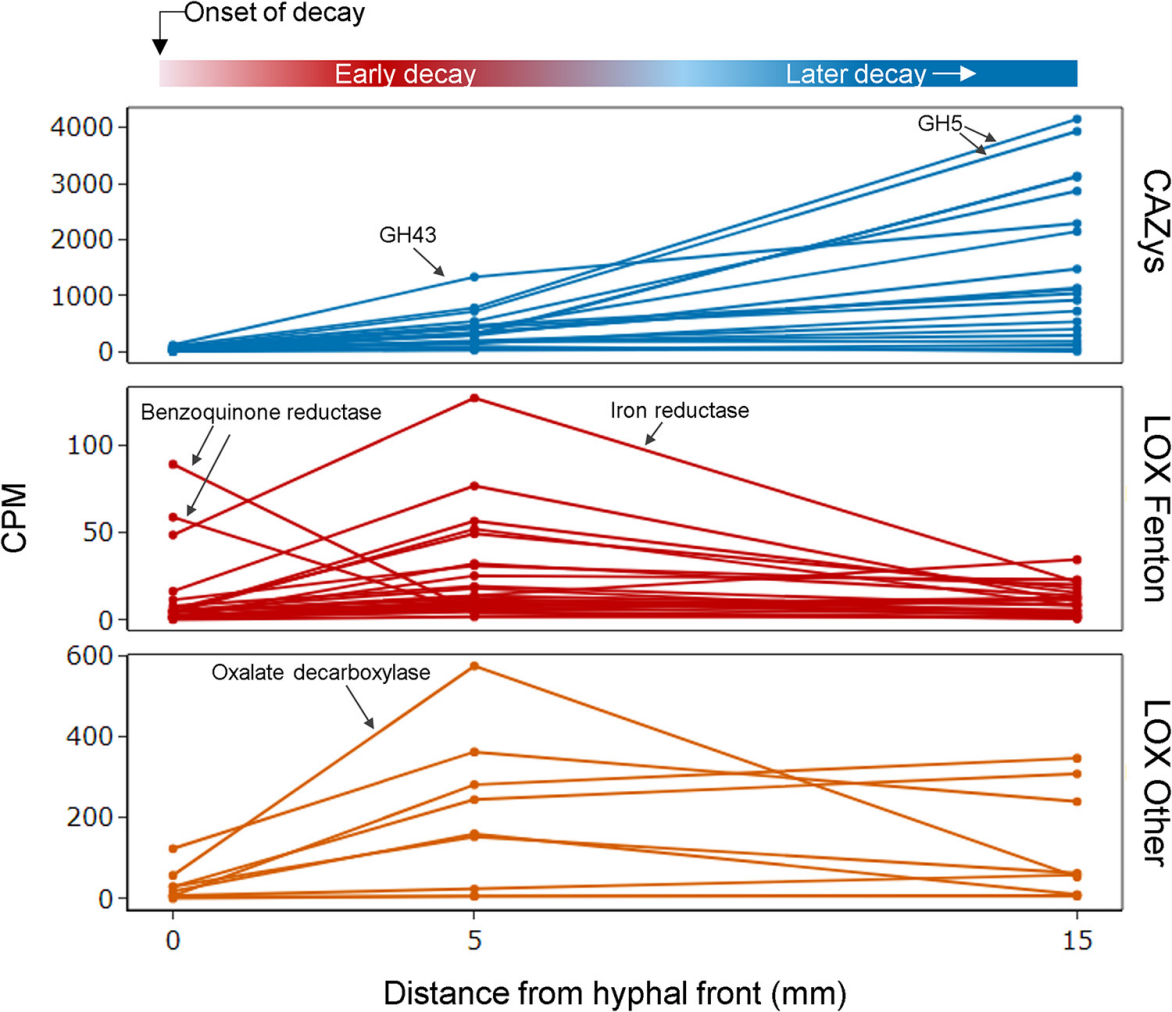
How short-term expression dynamics at the hyphal front fit within longer-term decay trends. *R. placenta* transcript investments in lignocellulose decay genes vary across early decay and a reference section at 15 mm behind the hyphal front. CAZys, represented here by key GHs (GH5, -12, -27, -43, and -51), have delayed expression within the first 5 mm and continue to ramp up into later decay stages. Most LOX genes, while delayed at the onset, still peak in the early decay stage. Conversely, benzoquinone reductase is upregulated in the first 100 μm of decay (same pattern for both alleles, as shown) and may play a key role in initiating brown rot decay. CPM, counts per million. Note the different scales on the *y* axes.

Delayed carbohydrate-active enzyme (CAZy) expression was recently noted as a defining characteristic of *R. placenta* and fungi in other brown rot clades (2–4). In this study, there were 92 CAZys with delayed expression, and none were incipiently expressed (Fig. 3). Among the 92 delayed CAZys were endoglucanases (e.g., glycoside hydrolase 5 [GH5] and GH12), the primary cellulases in *R. placenta* and most brown rot fungi. Also delayed were CAZys that typically have early upregulation when sampled at a lower resolution, including side chain hemicellulases (e.g., GH27, GH43, and GH51) and pectinases (e.g., GH28), both of which are often implicated in early depolymerization (Fig. 4).

### Lignocellulose oxidation genes are *“*off*”* at the hyphal front, with one key exception: benzoquinone reductase.

Previous studies of the brown rot mechanism show the importance of Fenton chemistry in the earliest stages of brown rot decay and generally imply that lignocellulose oxidation (LOX) genes are constitutively expressed (2, 15). However, the fine resolution of this study revealed that most LOX DEGs were delayed, including (i) those that generate extracellular Fe^2+^ (iron reductase) or H_2_O_2_ (alcohol oxidase and GMC oxidoreductase), (ii) those that have been implicated in hydroquinone metabolism (phenylalanine ammonia-lyase), and (iii) those that regulate oxalate concentrations (oxalate decarboxylase) (Fig. 3) (16–20). In notable contrast, there were two alleles of the benzoquinone reductase gene that were incipiently expressed (Fig. 3 and 4) (protein identifiers 64069 and 124517 from the Joint Genome Institute [JGI] database, Postia placenta MAD 698-R v1.0). Quinone reductases have been hypothesized to reduce a quinone to its hydroquinone, which can reduce both iron and oxygen (Fe^3+^ and O_2_) to generate the reactants required for the Fenton reaction (Fe^2+^ and H_2_O_2_) (8, 9, 21–24). Other non-LOX, decay-related DEGs were also incipiently expressed, including 2 citrate synthases and a cytochrome P450. The incipiently expressed DEGs in *R. placenta*, in particular the benzoquinone reductase, may have a major implication for the control of ROS by this fungus and possibly other brown rot fungi.

## DISCUSSION

We captured a major brown rot induction event, *in planta*, by evaluating fungal differential gene expression at a 50-fold-better resolution than previously observed. This was achieved by cryosectioning colonized wood and then using transcriptome sequencing (RNA-seq) to create a spatial sequence of decay and a finer-resolution map of fungal gene expression. Until now, early-upregulated genes have appeared as though they were “on” from the outset of brown rot. Here, only a small number of genes were incipiently expressed, and 21-fold more genes had delayed expression (Fig. 2), reflecting an inducible brown rot mechanism coincident with ROS production.

The genes showing delayed expression include side chain hemicellulases, pectinases, and endoglucanases. While the endoglucanases are known to be upregulated in later decay as step 2 of brown rot, the side chain hemicellulases and pectinases are typically upregulated earlier, even tolerating oxidative environments to facilitate the early removal of hemicellulose side chains that likely serve as early energy sources for the fungus (25, 26). In addition, 32 genes with lignocellulose oxidative functions were also delayed (Fig. 3; see also Data Set S1 in the supplemental material). These results suggest that either a time lag is required to activate most decay-related enzymes after hyphal tips first encounter the wood environment or a diffusible compound released from wood is inducing gene expression.

We discovered, however, that there was one intriguing exception to this early inducible pattern: a well-known and often-implicated participant in lignocellulose oxidation. Benzoquinone reductase was incipiently expressed and then almost immediately downregulated (i.e., by hour 48). This quinone reductase (QRD) in *R. placenta* is likely responsible for completing the hydroquinone redox cycle and enabling Fenton reactant production (16, 18). Brown rot fungi employ a hydroquinone redox cycle during which hydroquinones are oxidized to semiquinones that subsequently act as reductants on Fe^3+^ or O_2_, resulting in fully oxidized quinones, Fe^2+^, and hydroperoxyl radicals/superoxides that dismutate to generate H_2_O_2_ (8, 9, 21, 22). The hydroquinones are regenerated from the quinones via QRDs. The importance of the hydroquinone redox cycle and QRDs has been noted in *R. placenta* previously (8, 18) as well as in Serpula lacrymans (23, 24) and more so in Gloeophyllum trabeum (9, 27–29). These three fungi represent three separately evolved clades of brown rot fungi with apparent convergence on quinones for iron reduction. A recent study compared two of these brown rot fungi, *R. placenta* and G. trabeum, with the white rot fungi Trametes versicolor and Pleurotus ostreatus, which also have genes that encode quinone reductases (4). Using a much lower 5-mm resolution to compare decay stages, Zhang et al. found that QRDs were upregulated early in both brown rot fungi as well as T. versicolor but were not differentially expressed in P. ostreatus (4). To this end, benzoquinone reductase appears to be a very good candidate for more detailed characterization to find what enables this unique brown rot mechanism.

The DEGs discussed in this study could theoretically be a consequence of subapical branching at later decay stages. However, the branching rate across sections is fairly uniform, reported in the supplemental methods of a similar wood wafer study (2). In addition, RNA stability could theoretically lead to “false positives” in incipiently expressed DEGs if degradation occurs between the front and the older sections of wafers. These caveats generally stem from physical limitations when working with hyphae growing in wood and may be more easily addressed as tools develop.

Overall, this information at such a fine scale, near the hyphal front of a wood decay fungus, reveals details that have implications on a larger stage. First, understanding what controls a carbohydrate-selective pathway to decompose such a recalcitrant lignocellulose as wood is very relevant in bioprocess engineering. This brown rot pathway spares lignin as a by-product, and it requires fewer genes to achieve nearly complete sugar extraction. We have winnowed the pool of genes and highlighted one quinone pathway that is now poised for deep exploration. Second, the same inducible pathways are a valid trait to incorporate into ecosystem biogeochemical predictions, specifically for global carbon budgeting. Understanding how these unique fungi react to a cue gives us better insight into the variables that shape their success and efficiency in nature.

## MATERIALS AND METHODS

### Wood wafer colonization by *R. placenta* and sample harvest.

Using *Rhodonia placenta* (MAD698R; ATCC 44394) maintained on malt extract agar, modified soil-block microcosms (1:1:1 mixture of soil-peat-vermiculite, with a 50% moisture content) containing birch “feeder” strips were inoculated and incubated in the dark at 26°C with 70% relative humidity (RH) until a fungal mat was formed (approximately 2 weeks). Aspen wafers (60 by 20 by 2.5 mm), cut with the cross section as the largest face and wood rays oriented across the shorter 20-mm dimension, were placed into microcosms such that *R. placenta* would grow vertically up the wafer. This wafer system has demonstrated reproducibility in fungal growth, superimposed gene expression patterns, and spatial alignment of the visual front with the internal hyphal front (2, 4, 20, 25, 30). After 26 days of incubation (in the dark at 26°C with 70% RH), wafers with horizontal hyphal fronts (*n* = 6) were harvested, and 2 samples (7 by 5 by 2.5 mm) were cut from the newest 5 mm of the fungal growth (0 to 5 mm). To maintain the orientation, a notch was cut out of the top corner of the hyphal front edge. This process was repeated at the 15- to 20-mm distance to harvest the reference point (Fig. 1 and 4).

Samples were prepared for cryosectioning by submerging the sample in a fixative solution (pure high-performance liquid chromatography [HPLC]-grade methanol containing 0.1% [vol/vol] Triton X-100) to stabilize RNA, followed by a gradual equilibration to optimum cutting temperature (OCT) medium (catalog number 23-730-571; Fisher Scientific) using 100% methanol, 70% (vol/vol) ethanol, 96% (vol/vol) ethanol, 100% ethanol, and 50% (vol/vol) 1× phosphate-buffered saline in OCT medium. Samples were then embedded in OCT medium and frozen at −80°C.

### RNA-seq, DEG analysis, and gene function assignments.

Previously embedded samples in OCT medium were cryosectioned on a Cryostar NX70 cryostat (Thermo Fisher, Kalamazoo, MI, USA) at 12 μm from the leading edge of the sample until the full span of approximately 100 μm was cut (0 to 100 μm or 15,000 to 15,100 μm for the reference point). This was replicated by rotating the specimen 180° and sectioning it again to collect the 4,900- to 5,000-μm section. The cryosectioned samples were transferred to a 1.5-mL microcentrifuge tube and kept frozen to ensure RNA integrity. The RNAs from the cryosectioned samples were isolated using an RNAqueous-Micro total RNA isolation kit (catalog number AM1931), followed by full-length cDNA synthesis using the SMARTer Ultra Low RNA kit for Illumina sequencing (catalog number 634936). The cDNA was validated using the Agilent 2100 bioanalyzer, which indicated that the extracted RNA samples were in good condition to move forward with template library preparation. A Nextera XT DNA library preparation kit (catalog number FC-131-1096) and IDT for Illumina DNA/RNA UD indexes, set A, tagmentation (catalog number 20027213), were used to generate the template library for sequencing according to the manufacturer’s protocol. Single-read sequencing of the cDNA libraries with a read length of 150 bp was performed on the NextSeq 500 sequencing system using the NextSeq 500/550 high-output v2 kit, 150 cycles (catalog number 20024907).

Read trimming was conducted using bbduk (http://jgi.doe.gov/data-and-tools/bb-tools/) with parameters qtrim=rl, trimq=10, minlen=100, and maq=10. Reads, averaging over 27 million reads per sample, were aligned to the *Postia placenta* MAD 698-R v1.0 genome (https://genome.jgi.doe.gov/portal/pages/dynamicOrganismDownload.jsf?organism=Pospl1) (16) using Bowtie2 (http://bowtie-bio.sourceforge.net/bowtie2/index.shtml) with parameters –local –sensitive-local (31), resulting in an average 80.2% alignment. The “NH” field of multimapping reads in the SAM files was edited to 1, allowing multimapping reads to be randomly assigned to one mapping locus. This procedure allows duplicated/paralogous genes to be represented in the data. Read quality was assessed using FastQC (https://www.bioinformatics.babraham.ac.uk/projects/fastqc/) (32). Aligned reads were mapped to genes using htseq-count (33) with parameters -a 1, -t “exon” -i “name.” Differential gene expression was assessed using the R package DESeq2, which performs a Wald test on normalized counts (34). The 0- to 100-μm and 4,900- to 5,000-μm sections were analyzed as pairs within each wafer, with one or two pairs used from each wafer providing a total of 8 pairs. To maintain consistency with previous recent work with DEG profiling of this fungus, functional assignments of lignocellulose-degrading genes were adopted from those reported previously by Zhang et al. (2). An internal control gene (JGI protein identifier 125371), as recommended for *in planta* studies with *R. placenta* (35), was not differentially expressed under our criteria; however, there was some variability in the CPM (counts per million) values across sections, which is likely related to the small physical sample size.

### Data availability.

Data are deposited at the Gene Expression Omnibus database under accession number GSE193915.
